# House design modifications reduce indoor resting malaria vector densities in rice irrigation scheme area in western Kenya

**DOI:** 10.1186/1475-2875-8-108

**Published:** 2009-05-19

**Authors:** Harrysone Atieli, Diana Menya, Andrew Githeko, Thomas Scott

**Affiliations:** 1 Kenya Medical Research Institute, PO Box 1578, Kisumu, Kenya; 2 Moi University, School of Public Health, PO Box 4606, Eldoret, Kenya; 3 Department of Entomology, University of California, Davis, California 95616 USA

## Abstract

**Background:**

Simple modifications of typical rural house design can be an effective and relatively inexpensive method of reducing indoor mosquito vector densities and consequently decreasing malaria transmission. Public health scientists have shown the potential for house design to protect people against malaria, yet this type of intervention remains virtually ignored. A randomized-controlled study was, therefore, undertaken to determine the effects of this method of vector control on the density of indoor resting malaria vectors in a rice irrigation scheme area in lowlands of western Kenya.

**Methods:**

Ten treatment houses were modified with ceilings of papyrus mats and insecticide-treated netting (ITN) and tested against ten control houses without papyrus ceilings. To determine densities of mosquitoes resting in homes, the pyrethrum spray method was used to simultaneously collect indoor resting malaria vectors in intervention and control houses. Each house was sampled a total of eight times over a period of four months, resulting in a total of 80 sampling efforts for each treatment. Community response to such intervention was investigated by discussions with residents.

**Results:**

Papyrus mats ceiling modification reduced house entry by *Anopheles gambiae s.l *and *Anopheles funestus *densities by between 78–80% and 86% respectively compared to unmodified houses. Geometric mean density of *Anopheles gambiae s.l*. and *Anopheles funestus *in modified houses were significantly lower (t_18 _= 7.174, P < 0.0001 and t_18 _= 2.52, P = 0.02, respectively) compared to controls. Unmodified houses were associated with relatively higher densities of malaria vectors. There was a 84% (OR 0.16, 95% CI 0.07–0.39, P < 0.0001) and 87% (OR 0.13, 95% CI 0.03–0.5, P = 0.0004) reduction in the odds of *Anopheles gambiae s.l*. and *Anopheles funestus *presence in modified houses, respectively, compared with unmodified houses. Residents responded favourably to this mode of vector control.

**Conclusion:**

House modifications involving insect screen ceilings made from locally available materials and small ITN incorporated in house construction have the potential to reduce human exposure to malaria vectors, and thus parasite infection, in a rice irrigation scheme area of western Kenya. Ceiling modification is likely to be acceptable and is expected to be of greatest benefit when used in combination with other malaria control strategies.

## Background

Malaria control in the tropics is currently based largely on treatment of people with clinical illness and personal protection against malarial mosquito vectors. Vector control campaigns emphasize environmental sanitation and suitable environmental management, implementation of educational programs and the use of insecticides, either in impregnated fabrics (i.e., mosquito nets and curtains) or sprays (indoors and outdoors). The use of pesticides, which has to be highly regulated and their handling is subject to strict control, requires adherence to the recommendations of the WHO [1,2]. Scant consideration has been given to housing design and construction as an environmental strategy to control malaria [3].

Insecticide-treated bed nets can be highly effective for controlling malaria [4]. However, in the high transmission areas, additional measures will be needed to reduce the malaria burden to acceptable levels. Poor quality housing is generally accepted to be an important contributor to ill health [5]. In most parts of Africa, feeding by the principal malaria vectors, *Anopheles gambiae *and *Anopheles funestus*, takes place in the later part of the night, indoors [6]. As a result, entry rates and hence malaria transmission are affected by house construction. Thus, improved housing construction is regarded as among the main methods to control malaria since early 20^th ^century [3]. Although the importance of housing for health is recognized [5,7,8], few well-designed studies have quantified its impact, especially in the developing world.

Previous studies in a malaria endemic community in Sri Lanka [9] found a strong association between malaria incidence and the type of house construction, independent of the house's location and residents' behavioural patterns. The risk of getting malaria was greater for inhabitants of the poorest type of houses, characterized by incomplete construction with thatched roofs and walls made of mud or cadjan (woven coconut palm leaves), compared with better-constructed houses with complete brick and plastered walls and tiled roofs. A significantly higher number of indoor resting mosquitoes were found in the poorly constructed houses than in the better-constructed ones. In a later study in the same area, the risk of malaria was found to be 2.5-fold higher for people living in poorly constructed houses than for those living in houses of good construction [10]. In Tanzania, houses built of bricks and with zinc roofs are associated with lower levels of malaria-associated anaemia compared to poorly built mud-walled houses [11]. Similarly, in rural Gambia, installing ceilings or closing eaves in experimental huts was found to protect people from malaria mosquitoes [3]. Being endophilic and endophagic [6], all efforts done to reduce entry of malaria vectors through improved house construction, in particular screening against mosquito entry, has long been recommended as a way of reducing human exposure to mosquito bites and infection with malaria parasites [12].

Insect behaviour can be exploited when designing control strategies for control of disease vectors and other pests. *Anopheles gambiae*, the main malaria vector in Africa [6], is well-adapted for entering houses, because it flies upwards when encountering a vertical surface [13]. Attracted to human odours emanating from a house, many *Anopheles gambiae *reach an outside wall and fly up, funneled indoors by the overhanging roof of the typical village house design, through the open eaves [3,14]. This mode of house entry suggests that closing eaves or installing ceilings would give protection [3,12]. This implies that houses with open eaves, or which lack ceilings, are associated with increased numbers of mosquitoes and higher levels of malaria compared with neighbouring houses with closed eaves or ceilings [12]. The objective of this study, therefore, was to determine the effect of ceilings made from locally available traditional building materials as barriers against house-entry by malaria vectors in an endemic rice irrigation scheme area in the lowlands of western Kenya.

## Methods

### Study area

**Figure 1:**
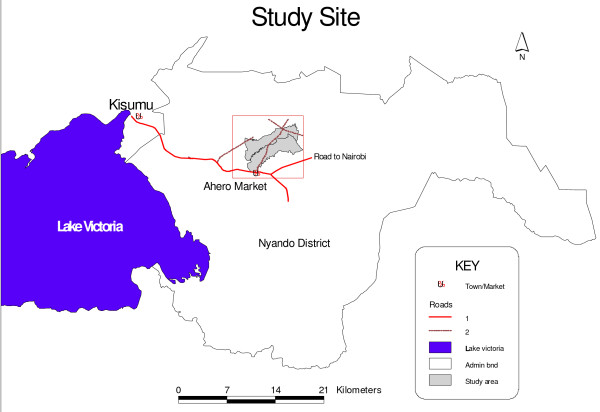
**Map of western Kenya showing location of study site in Ombeyi division, Nyando district**.

Our study was carried out in Kore and Ahero irrigation scheme sub-locations in Ombeyi division of Nyando district (Figure 1). It is located 24 km southeast of Kisumu in Nyanza province in western Kenya, 15 km south of the Equator at an altitude of 1,150 m above sea level (latitude 34.90E and 34.97E and longitude 0.11S and 0.16S). It covers an area of 29 km^2 ^and has a population of about 7,891 people [15]. Annual mean temperatures vary between 17°C and 32°C. The area is relatively humid due to its proximity to Lake Victoria. Local climate is characterized by three peaks of rains with an average annual rainfall of 1,000 – 1,800 mm and an average relative humidity of 65%. The first peak of rains occurs between March and July, with an average monthly rainfall of 150 – 260 mm. The other rainy season occurs in August. Short rains occur between September and October and have an average monthly rainfall of at least 125 mm. The dry period occurs between December and February. The main economic activities include rice irrigation, subsistence farming, cultivating maize, sorghum, cassava, millet, and vegetables. Many people keep animals including cattle, goats, sheep, and poultry. Other activities include fishing due to its proximity to Lake Victoria and the Nyando River. There is local marketing of food and grain to the nearby small town of Ahero and to Kisumu. Some villagers commute daily to work in Kisumu city. Study villages were selected because they overlapped with the area used as the focus of other related studies, making possible the use of existing baseline information. Previous studies in the area show that most of the houses are typically constructed of a stick framework with mud walls and thatch or corrugated metal roof [16].

Malaria is highly endemic in the region. Transmission occurs throughout the year. The mean annual *Plasmodium falciparum *sporozoite inoculation rates range from 90 to 410 infective bites per year [16,17]. The principal mosquito vectors in the area are *Anopheles gambiae*, *Anopheles funestus *and *Anopheles arabiensis *[18]. Of the three malaria vectors, *Anopheles gambiae s.l*. and *Anopheles funestus *are highly endophagic and anthropophagic, with a mean of seven adult females collected per house [19].

### Papyrus mats

**Figure 2:**
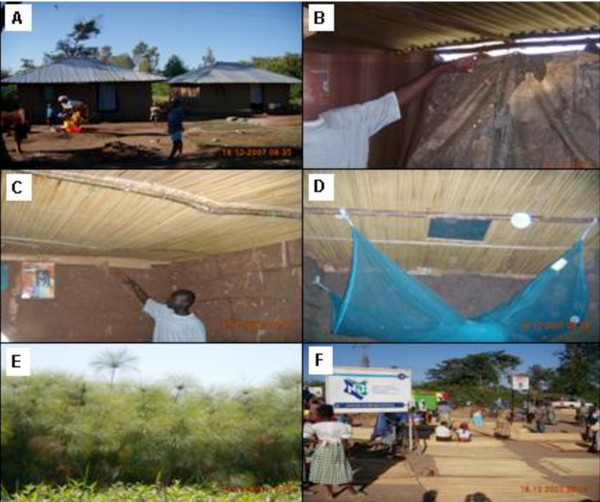
**Study houses and modifications**. A) Typical village house with funnel shaped roof; B) Open eave between roof and wall; C) Closed eaves with papyrus mats ceiling; D) Sleeping room with mat ceiling, small ceiling ITN and bed net; E) Papyrus reeds in nearby swampy farm; F) Mats being sold at nearby Ahero market.

Community members and womens groups within the study location were asked to weave ceilings from papyrus reeds. They harvested papyrus reed stalks from nearby swamps and left to dry for a week. Stalks were weaved into mats of about 2 by 3 metres, using sisal strings, which are locally and readily available. Mats were bought from these groups at market price (one dollar per mat). Once weaved, these mats were fixed in intervention study houses entire roof space below the open eaves (Figure 2).

### Entomologic collections

From early October 2007 to the end of February 2008, indoor-resting mosquito densities were determined based on the pyrethrum spray collection (PSC) method [20]. There were 80 sampling occasions each for treatment and control houses at two week intervals during the study period. Before a pyrethrum spray collection from a selected house, a member of the household was asked about the number of people who had slept there the previous night. A team of one entomologist and two assistants collected mosquitoes from the house. After all exits had been covered, a white cotton sheet was placed on the floor, and a pyrethrum-based un-residual insecticide was sprayed in the room. Ten minutes later, the dead mosquitoes were collected from the sheet, stored in separate individual vials for each house and kept in a cooler box. All mosquitoes collected were transported back to the laboratory at Kenya Medical Research Institute, identified with a microscope on the same day and recorded by species, gender, and by degree of blood meal engorgement status for females.

### House characteristics, distribution and location

Before randomly selecting study houses for entomologic sampling, the exact location of all houses in the two villages was determined with a hand-held global positioning system receiver (e Trex HC series, Garmin International, Inc). A unique identification number was clearly marked on the door beam of the entrance to each house. This information was used to establish a geographic information system (GIS) for study houses using ArcView software, which made it possible to randomly select study houses using random number selection method. Randomly selected study houses were typical traditional semi permanent defined as having a stick framework, mud walls and iron sheet roofs. They had open eaves with at least three regular sleepers. Selected houses were replaced with the nearest neighbour house, if they did not fulfil the above criteria.

### Netting impregnation with permethrin

Permethrin extract Icon 10 WP was obtained from the pyrethrum board of Kenya, Nakuru. Before nets were impregnated, the amount of water they retain at saturation was determined by measuring the amount of water left after dipping a net of known area in a known volume of water. The amount of water absorbed by 1 m^2 ^netting material was used to calculate the volume of insecticide required for impregnation of a given area of netting at a rate of 0.5/m^2^. Once impregnated, netting was left to dry in a shaded area before fixing it in a 1.5 feet ceiling opening above the sleeping room (Figure 2).

### Social science

Key informant discussions about the use, acceptance, affordability and sustainability of this locally available and relatively cheap traditional building material as ceilings for malaria vector control were held with village leaders, masons, carpenters and home owners. Separate group discussions were also conducted with men and women of almost similar age so that participants did not feel inhibited to freely comment on the subject.

### Meteorological measurements

Temperature and humidity in five houses of each treatment was recorded throughout the study period using temperature-humidity loggers (Onset computer corporation, EU), with the assumption that papyrus reeds ceilings would provide insulation from heat generated by iron sheet roofs.

### Data management and statistical analysis

Exposure to malaria vectors in the study houses was estimated to be the sum of mosquitoes collected from control versus intervention houses. Data were analyzed with STATA SE 9 (Stata Corporation, USA) and JMP 5.0.1  software. Differences in mosquito densities in modified houses and unmodified houses were determined using a t-test and multiple testing corrections by repeated measure ANOVA because the same houses were sampled multiple times (eight times) resulting in non-independence data points. Chi-square tests were done to determine the odds of association of house type with the presence of malaria vectors. Residents' response on the use, accessibility, affordability and sustenance of house design modification as a method of vector control was determined following informed consent through focus group discussions.

### Ethical clearance

The protocol for this study was approved by the Institutions Review and Ethics Committee (IREC) of Moi Teaching and Referral Hospital and Moi University (Reference: IREC/2007/41, Approval Number: 000258).

## Results

### House characteristic and malaria vector abundance

**Figure 3:**
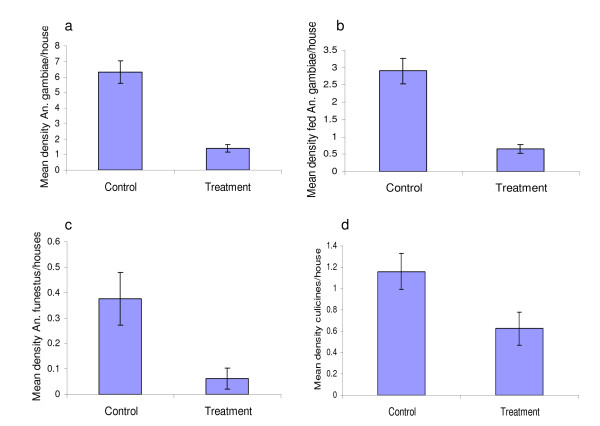
**Mean number of mosquitoes entering houses, a) Mean density of female *Anopheles gambiae s.l*/house; b) Mean density of fully fed *Anopheles gambiae s.l*/house; c) Mean density of *Anopheles funestus*/house; d) Mean density of *Culicines*/house**.

There were statistically significant differences between the number of *Anopheles gambiae s.l*. ((t_18 _= 7.174, P < 0.0001) and *Anopheles funestus *(t_18 _= 2.52, P = 0.02) collected in control and treatment houses. Figure 3 shows differences in mean density of indoor resting mosquitoes caught in either modified or unmodified houses during the study period. Of the 793 female mosquitoes collected from October 2007 to March 2008, 615 (77.6%) were *Anopheles gambiae s.l*., 35 (4.4%) *Anopheles funestus *and 143 (18%) culicines. Most *Anopheles gambiae s.l*., 501 (81.5%) were collected in control houses, only 114 (18.5%) came from modified houses. For *Anopheles funestus*, 30 (85.7%) and 5 (14.3%) were collected in control and modified houses, respectively. For Culicines, 93 (65%) and 50 (35%) were collected in control and modified houses, respectively. Due to their larger sample size, in further analyses only *Anopheles gambiae s.l*. were considered (Table 1).

**Table 1:** Protective efficacy of house design modifications against house-entering malaria vectors

	*Anopheles gambiae*				
Survey Date	No. collected in control houses	No. collected in modified houses	Mean number of mosquitoes in control houses (95% CIs)	Mean number of mosquitoes in modified houses (95%CIs)	Percentage reduction compared with control

10/22/07	37	12	4(2–6)	1(1–3)	68
11/05/07	41	10	4(2–6)	1(-1–3)	76*
11/21/07	66	14	7(4–9)	1(-1–4)	79*
12/03/07	55	11	6(3–8)	1(-1–3)	80*
12/18/07	75	15	7(2–12)	1(0–2)	82*
02/05/08	66	16	7(2–12)	2(-0–4)	76*
02/21/08	77	19	8(1–15)	2(0–4)	76*
03/06/08	84	17	8(3–14)	2(1–3)	80*

CI, confidence interval, * P < 0.05, Degree of freedom 18 (n-2) for all surveys dates, No, Number.

*Anopheles gambiae *and *Anopheles funestus *were significantly less abundant in modified than unmodified houses, thus unmodified houses were associated with significantly elevated densities of malaria vectors. Overall, there was a 84% (OR 0.16, 95% CI 0.07–0.39, P < 0.001) *Anopheles gambiae s.l*. reduction and 87% (OR 0.13, 95% CI 0.03–0.05, P < 0.001) *Anopheles funestus *reduction in the odds of their presence in modified houses compared with unmodified houses, which were characterized by open eaves with neither ceiling nor fixed ITN on the ceiling. Houses with open eaves also tend to have statistically significant higher mean densities of fed *Anopheles gambiae *than modified houses (t_18 _= 6.178, P < 0.0001) (Figure 3). Modification likewise significantly reduced Culicines densities by 46% (t_18 _= 2.08, P = 0.053).

There were significantly fewer *Anopheles gambiae s.l*. in houses modified with papyrus ceilings compared with the controls in all sampling periods (P < 0.05, Table 1) except the first sampling, which narrowly missed statistical significance (t_18_= 1.73, P= 0.0503). A papyrus ceiling reduced the number of *Anopheles gambiae s.l*. entering huts by 76–82% (P < 0.001) compared with controls. Very few *Anopheles funestus *and Culicines were collected (0.2 and 0.9 respectively) but, similarly, ceiling modification resulted in a statistical significance reduction on *Anopheles funestus *mean densities by 86% (t_18 _= 2.52, P = 0.02) and Culicines by 46% (t_18 _= 2.08, P = 0.053). Table 1 shows protective efficacy of ceilings made of papyrus mats against house entering *Anopheles gambiae s.l*. during different survey periods.

### Effect of modification on indoor temperatures and humidity

**Figure 4:**
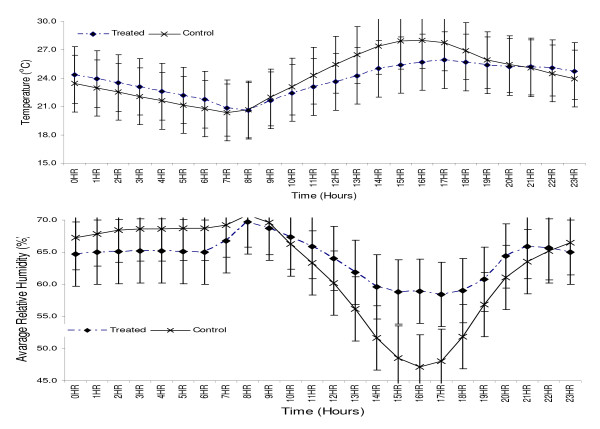
a) Twenty four hour average indoor temperatures, b) twenty four hour average relative humidity.

The presence of a papyrus ceiling and ITN modification had no statistical significant difference on indoor temperature and humidity. However, they were generally associated with cooler indoor temperatures during the day and warmer during the night compared to unmodified houses with open eaves. Papyrus mats ceiling modified houses had an average cooler indoor temperature of 1.3°C during the day compared to unmodified houses. During the night, modified houses were warmer by an average 0.8°C. The largest cool difference in temperature (2.6°C) during the day between modified and unmodified houses was recorded at 3 pm. Warmer night time temperature difference of 1°C occurred between 1 am-6 am. Modified houses had an average higher humidity (62.9%) than unmodified houses (57.8%) during the day. At night, unmodified houses had relatively higher humidity (67%) than modified houses (65.3%), but with fewer differences during the day (Figure 4).

### Community response on mode of vector control

Individual and group interviews were conducted with house owners, village elders and traditional house constructors. All adult interviewees had knowledge of malaria and indicated mosquitoes as its possible vector. Most community members favourably accepted papyrus ceilings as a relatively cheaper method of malaria vector control. There were no significant differences in the response given by spouses. Responses were remarkably consistent, demonstrating a general perception that ceilings played an important role in vector control and improve the general functionality and beauty of houses. Most houses (90%) in the overall study area had no ceilings or closed eaves. Ninety two percent (n = 123) cited cost of importing soil for house construction and high daytime temperatures as main reasons for leaving eaves open. Extra effort required in weaving papyrus mats and incorporating them in house construction were cited as the main reasons for not including papyrus ceilings in houses.

## Discussion

There is need for those keen to control malaria in the tropics not to forget a lesson from the past; i.e., that improvements in living conditions can reduce malaria [3]. Unfortunately, quite often inadequate attention is given to changes in house design as a contributing factor to reduction of malaria in many parts of the world.

In this study, we demonstrated that addition of a simple ceiling with insecticide impregnated netting fixed above the sleeping room of traditionally designed houses can substantially reduce human exposure to mosquito vectors of malaria. The ceiling modification reduced house entry by *Anopheles gambiae s.l*. by about 76–82%. This degree of protection compares well with the 86–91% and 80% reduction in biting seen with insecticide-treated bed nets and different ceiling materials, respectively, reported from earlier studies using experimental huts [3,21,22]. The small ITN fixed in the sleeping room ceiling acted as a decoy trap. Presumably, host odours from the room pass up through the netting and out the eaves, attracting mosquitoes into the roof space, where they were prevented from entering the room by the ceiling and are killed when they contact the ITN. Previous studies have shown, depending on the material used and the resistance profile of local mosquitoes, that the protection afforded by such insecticide treated netting does not repel mosquitoes from entering via the door or window [3]. This qualifies the fact that differences in vector densities between control and intervention houses were dependent on the condition of eaves. If, however, insecticide was applied to curtains around the door and windows it is likely that protection would be further improved. Treating curtains with permethrin at a village scale in Burkina Faso was associated with a reduction in malaria vectors by about 99.5% [23-26]. On the other hand, in places where people are reluctant to use bednets because there are few nuisance mosquitoes [27,28], ceilings or closed eaves could be an effective alternative method of protection.

Previous studies at Ahero indicate that more than 90% of villagers are indoors and in their beds by 2100 hr. Biting activity is low before this time. The risk of getting bitten increases in the later hours of the night [16]. Modified ceilings provide protection for the entire family against bites from malaria vectors, compared to bednets which protect only the person or persons sleeping under the net. Through interviews and surveys, we determined that almost all traditional house designs have open eaves because importing soil to fill in the opening is too costly and eaves provide ventilation against high indoor temperatures during the day. Permanent houses had closed eaves and fixed ceilings. This indicates that due to house design inhabitants of traditional houses are at a higher risk of being bitten by malaria infected mosquitoes than are people who live in permanent houses [29].

Although not statistically significant, papyrus reeds mats ceilings did not make rooms hotter, but they did reduce evaporation by about 2–5%. Unlike previous studies in which different ceiling materials were used [3], our modification did not reduce air flow or make rooms feel stuffy. Our design allowed air circulation through mats and the ITN window, thus increasing acceptability of papyrus ceilings to home owners. Modified houses were noticeably cooler (-1.3°C) during the day and warmer (+0.8°C) during the night. This is consistent with the insulation effect of the ceiling to heat from iron sheet roofs and heat from living room during the day and night respectively. Results from focus group interviews indicate that ceilings were cheap, considered to beautify houses, widely associated with reduced temperatures and less associated disturbance by mosquitoes, all of which support the notion that installing ceilings to reduce entry by mosquitoes would likely receive broad community support. Using readily available traditional building material (papyrus reeds) for ceiling construction was inexpensive. It costs about a dollar/person; assuming a ceiling life of over 10 years and three people per house. The only out of pocket costs are for screening and insecticide.

Control measures against mosquito bites are expected to have a beneficial impact by reducing malaria morbidity and mortality [30]. The degree of control needed to obtain a particular public health impact would clearly vary among sites, and control strategies should take the initial transmission levels into account. For example, there is evidence from The Gambia that communities with the lowest vector densities are at greatest risk of disease [28]. Our study shows that ceilings with an insecticide treated netting window can substantially reduce human exposure to malaria vectors. It is likely that the greatest effect on clinical malaria would be seen in areas of moderate and high transmission where a reduction in exposure to malaria parasites would lead to a proportional reduction in morbidity. Based on results from our study we recommend that intervention trials that measure epidemiological outcomes should be conducted in areas of moderate to high transmission to determine the protective efficacy of ceilings against clinical malaria.

## Conclusion

Results from this study demonstrate that the domestic environment can be altered in novel ways to reduce the human-vector contact rate. House design modification, such as modified ceilings, should be considered as part of an integrated approach in malaria vector control. Improvements to the domestic environment can discourage or prevent entry of anthropophagic mosquitoes into homes. Reduction in the number of mosquitoes indoors is expected to lead to a reduction in the number of bites received per occupant per year, which in turn would reduce malaria incidence. The domestic environmental factor we identified, i.e., open eaves, is a risk factor that should be further studied to determine what additional modifications can be made to existing homes by their occupants utilizing readily available materials to reduce the number of mosquitoes in homes.

## Competing interests

The authors declare that they have no competing interests.

## Authors' contributions

HA designed and implemented the study. He did the data analysis and wrote the manuscript. DM participated in the design of the study. AG and TS participated in the design and coordination of the study and writing of the manuscript.

## References

[B1] WHO (2004). Frequently asked questions on DDT use for disease vector control EHO/HTM/RBM/200454.

[B2] WHO. RBM; 2001–2010 (2002). United Nation Decade to Roll Back Malaria. Geneva.

[B3] Lindsay SW, Jawara M, Paine K, Pinder M, Walrave GE, Emerson PM (2003). Changes in house design reduce exposure to malaria mosquitoes. Trop Med Int Health.

[B4] Lengeler C (2004). Insecticide-treated bed nets and curtains for preventing malaria. Cochrane Database Syst Rev.

[B5] United Nations Centre for Human Settlements (1996). An urbanizing world: global report on human settlements.

[B6] Gillies MT, De Meillon B (1968). The Anopheline of Africa South of the Sahara. Publication No 54.

[B7] United Nations Habitat II Conference (1996). Report of the United Nations conference on human settlements (habitat II) Istanbul.

[B8] WHO (1998). Roll Back Malaria. World Health Organisation.

[B9] Gamage-Mendis AC, Carter R, Mendis C, de Zoysa APK, Herath PRJ, Mendis KN (1991). Clustering of malaria infections within an endemic population: risk of malaria associated with the type of housing construction. Am J Trop Med Hyg.

[B10] Gunawardena DM, Wickremasinghe AR, Muthuwatta L, Weerasingha S, Rajakaruna J, Senanayaka T, Kotta PK, Atan (1998). Malaria endemic regions of Sri Lanka, and the impact and cost implications of risk factor-based interventions. Am J Trop Med Hyg.

[B11] Kahigwa E, Schellenberg D, Sanz S, Aponte JJ, Wigayi J, Mshinda H, Alonso P, Menendez C (2002). Risk factors for presentation to hospital with severe anaemia in Tanzanian children: a case-control study. Trop Med Int Health.

[B12] Lindsay SW, Emerson PM, Charlwood JD (2002). Reducing malaria by mosquito-proofing houses. Trends Parasitol.

[B13] Snow RW, Jawara M, Curtis CF (1987). Observations on *Anopheles gambiae *Giles s.l (Diptera Culicidae) during a trial of permethrin treated bed nets in the Gambia. Bull Entomol Res.

[B14] Githeko AK, Service MW, Mbogo CM, Atieli FK, Juma FO (1994). Sampling *Anopheles arabiensis*, *A. gambiae sensu lato *and *A. funestus *(Diptera: Culicidae) with CDC light traps near rice irrigation area and a sugar cane belt in western Kenya. Bull Entomol Res.

[B15] Housing and Population Census of Kenya (1999). Housing and population Census of Kenya.

[B16] Githeko AK, Adungo NI, Karanja DM, Hawley WA, Vulule JM, Seroney IK, Ofulla AV, Atieli FK, Ondijo SO, Genga IO, Odada PK, Situbi PA, Oloo JA (1996). Some observations on the biting behavior of *Anopheles gambiae *s.s., *Anopheles arabiensis*, and *Anopheles funestus *and their implications for malaria control. Exp Parasitol.

[B17] Beier CJ, Perkins PV, Onyango FK, Gargan TP, Oster CN, Whitmire RE, Koech DK, Roberts CR (1990). Characteristics of malaria transmission by Anopheles (Diptera: Culicidae) in western Kenya in preparation for malaria vaccine trials. J Med Entomol.

[B18] Fontaine RE (1978). House spraying with residual insecticides with special reference to malaria control. WHO/VBC/78704.

[B19] Githeko AK, Service MW, Mbogo CM, Atieli FK, Juma FO (1994). Origin of blood meals in indoor and outdoor resting malaria vectors in western Kenya. Acta Trop.

[B20] WHO (1975). Manual on practical entomology in malaria. Part II: Methods and Techniques. Division of Malaria and other Parasitic Diseases.

[B21] Lindsay SW, Adiamah JH, Miller JE, Armstrong JRM (1991). Pyrethroid-treated bednet effects on mosquitoes of the *Anopheles gambiae *complex in the Gambia. Med Vet Entomol.

[B22] Miller JE, Lindsay SW, Armstrong JR (1991). Experimental hut trials of bednets impregnated with synthetic pyrethroid or organophosphate insecticide for mosquito control in The Gambia. Med Vet Entomol.

[B23] Procacci PG, Lamizana L, Kumlien S, Habluetzel A, Rotigliano G (1991). Permethrin-impregnated curtains for malaria control. Trans R Soc Trop Med Hyg.

[B24] Procacci PG, Lamizana L, Pietra V, Di Russo C, Rotigliano G (1991). Utilization of permethrin-impregnated curtains by the inhabitants of a rural community in Burkina Faso. Parassitologia.

[B25] Pietra Y, Procacci PG, Sabatinelli G, Kumlien S, Lamizana L, Rotigliano G (1991). Impact de I'utilisation des rideaux imprégnés de permethrine sur le paludisme dans une zone rurale de haute transmission au Burkina Faso. Bull Soc Path Exot.

[B26] Habluetzel A, Cuzin N, Diallo DA, Nebie' I, Belem S, Cousens SN, Esposito F (1999). Insecticide-treated curtains reduce the prevalence and intensity of malaria infection in Burkina Faso. Trop Med Int Health.

[B27] Aikins MK, Pickering H, Alonso PL (1993). A malaria control trial using insecticide-treated bed nets and targeted chemoprophylaxis in rural area of The Gambia, West Africa. 4. Perception of the causes of malaria and its treatment and prevention in the study area. Trans R Soc Trop Med Hyg.

[B28] Clerke SE, Bogh C, Brown RC, Walraven G, Tthomas CJ, Lindsay SW (2002). Risk of malaria attacks in Gambia children increases away from malaria vector breeding sites. Trans R Soc Trop Med Hyg.

[B29] Yazoume Y, Moshe H, Louis V, Simboro S, Issouf T, Rainer S (2006). Housing conditions and *Plasmodium falciparum *infection: protective effect of iron-sheet roofed houses. Malar J.

[B30] Lengeler C (2004). Insecticide-treated bed nets and curtains for preventing malaria. Cochrane Database Syst Rev.

